# Medical and Physical Intelligence

**Published:** 1804-07-01

**Authors:** 


### MEDICAL AND PHYSICAL INTELLIGENCE.

### *Notice from the* VACCINE POCK INSTITUTION, *No.* 44, *Broad-Street, Golden-Square, June* 20, 1804.

THE public mind being of late much disturbed in consequence of successive reports during the whole of the last year, and especially of late, by publications of cases *esteemed* to be instances of the Small Pox two or three years subsequently to the Cow Pock, the Medical Establishment of this Institution have thought it their duty, whatever may be their own opinions, not to be inactive and silent.

Accordingly, I am directed to state, that in the last fortnight a number of subjects, who had undergone vaccination in the year 1800 (*the first year of the new practice at any professed institution,*) have been submitted to the test or counter proof, *variolation*, in circumcircumstances the most favourable for exciting the Small Pox.—Besides these trials, additional ones have been instituted in subjects who were vacinated in Dr. Pearson's early practice in 1799. Further,—Reports have been already received at the Institution from several provincial correspondents, who were witnesses to whole parishes of subjects vaccinated under Dr. Pearson's and Mr. Keate's inspection, or with matter furnished by them early in the year 1799.*

A very brief, but it is presumed, conclusive statement of evidence collected from these sources on the question, with which some persons have agitated the minds of so many families, is intended to be laid before the public in a week or ten days. This statement, it is apprehended, will be the most proper return to the respectable author,† who has lately addressed his pamphlet, "*To the Directors of the Vaccine Institution*," very justly conceiving that "the point at issue is within the power of this Institution, if they will give directions for a number of persons to be inoculated with Small-pox matter, or exposed strongly to infection, who were vaccinated early in the practice.

As no other professed Vaccine Institution but this has been established long enough to ascertain this demand, it has been determined to comply.

W. SANCHO, Secretary.

### *True Character of the Plague.*

To compensate for all the distresses brought on humanity by the campaigns in Egypt during the late war, it is consoling to reflect upon the discoveries which have been made by English and French physicians and officers, of the TRUE NATURE OF THE PLAGUE. Among all the writers of both nations, Affalini and Sir Robert Wilson deserve most credit. The latter disproves the old superstitious notion, that this disease is imported from Turkey into Egypt. He proves that it is the effect of exhalation from the putrid matters left by the overflowing of the Nile, and from other liquid and putrid matters in other parts of the country. He says it is communicated by the atmosphere, and that persons recover by being removed from the places where they are affected by it, to an air beyond

* It may be very important information to affirm, that the matter now used at this Institution was THAT originally taken in January and February, 1799, by one of the Physicians, from cows in Mary-le-Bone Fields and Gray's Inn Lane, with the addition, about three years ago, of matter from the Milanefe, by Dr. Sacco. But it does not appear that this extensive succession has at all altered the properties, nor that there is any difference of properties among these different sources of matter. The experience of this Institution does not justify the conclusions that the failure of Cow-pock in preventing the Small-pox depends in general upon the selection of matter on a particular day.

† See Mr. Goldson's Cases of Small-Pox subsequent to Vaccination, lately published.

beyond the reach of putrid exhalation. He denies its ever being contagious, except under those circumstances of filth and close confinement which now and then render all other fevers liable to become sources of infection. The general belief in these facts is calculated to do away the accumulated evils of ages, which have arisen from a belief of the plague being a specific disease, and kept alive like small-pox, only by being propagated from one person, or one country, to others. Millions of lives have been lost by this error. Sir Robert Wilson does not hesitate to declare, that the plague might be annihilated in Egypt, or made a mild disease; and mentions, as the means for that purpose, the use of lime, coals, paving the streets of their cities, the formation of roads, the white washing of the apartments in every house, the use of well-burnt brick instead of wood in their buildings, and the draining off all stagnant waters. He says, the most effectual remedies now employed for its cure are embrocation of the body with sweet oil, and the use of mercury.

---

### *Discovery of a new Vegetable Acid.*

Mr. KLAPROTH, on examining a saline substance, found on the bark of the white mulberry tree, (Morus alba L.) in the botanic garden of Palermo, by Mr. Thomson, discovered that it contained a new acid. The substance itself is of a browinsh colour, covers and penetrates the bark of the tree. Its taste resembles that of succinic acid. Thrown on burning coals, it puffs up and burns, leaving a terreous residuum; 100 parts of water dissolve 35 parts of it in the heat, and 15 parts in the cold. By evaporation, needle like radiant crystals are obtained. Barytes makes no precipitate in the solution of this salt. Alkaline carbonates however occasion a brown precipitate, the colour of which, by calcination, is changed into white. It is dissolved with effervescency in nitric acid. Sulphuric and oxalic acid occasion, in its solution in nitric acid, precipitates, which indicate the presence of lime. Accetit of lead forms in it an insoluble precipitate which was easily reducible on burning coals; nitrate of mercury formed white flakes, and nitrate of silver brown streaks. These experiments induced Mr. Klaproth to conclude that this salt, collected on the bark of the mulberry tree, is composed of lime and a particular vegetable acid. On decomposing this salt by the carbonate of ammonia, Mr. Klaproth obtained a precipitate of carbonate of lime. The liquor yielded by a proper evaporation, long straight prisms; and even the water of these crystals precipitated the nitric solutions of copper with a green colour of kobalt, pale red; of iron, brown; and of mercury, silver and lead, brown. The same liquor made water and acetite of lead slightly turbid, and likewise the muriates of tin and of gold, and the nitrate of nickel. In order to obtain this acid pure, Mr. Klaproth employed the precipitate, obtained from a mixture of a solution of the calcareous salt and acetite of lead. This precipitate was afterwards decomposed by diluted sulphuric acid. The proportions employed were forty-five grains of the precipitate and twenty-four grains of acid, diluted with one drachm of water. The sulphate of lead was separated by the filtrum. The liquor being evaporated, yielded by crystallization, thirty-four grains of acid in fine needles of a pale wood colour. The natural calcareous salt was also directly decomposed by sulphuric acid, and the result of which was the same. He employed thirty grains of the salt, and twelve of sulphuric acid. The properties of this new acid consist in its having a similar taste to succinic acid; in being exposed to the contact of air, without experiencing any change; in dissolving with ease in water and alkohol; in not precipitating metallic solutions. When distilled, part of it is destroyed, but the rest sublimed, which proceeding may be employed to separate it from the adhering extractive matter. Mr. Klaproth proposes to call it *moronitic acid*, and its saline combinations, *moronitates*.

---

M. Demmenie has noticed that the solution of copal may easily be effected by exposing it to the vapours of alcohol, or oil of turpentine. For that purpose an alembic may be fiilled one-fourth with either of these fluids, and some pieces of copal suffered to be suspended by threads in it, over the surface of the fluid. After having made the alcohol, or oil of turpentine, boil, the copal becomes liquified, and is dissolved.

---

The oliferous Chinese radish, the *raphanus Chinensis annuus oliferus*, is much cultivated in Piedmont and the Milanese. From $3\frac{1}{2}$ ounces of seed, a farmer, named Grandi, obtained a produce of 583 pounds, which yielded 200 pounds weight of oil. The Chinese extract from the seed half its weight in oil. It is employed by the Italians for culinary purposes, burns without emitting any smoke, and gives light as clear as common oil. In the Milanese, the seed is sown in March, the land having been ploughed in autumn, and again before the seed is sown, but not manured. The plants are to be thinned to the distance of three or four inches from each other.

---

An experiment has lately been made at Lyons to try the effects of vaccination in preserving fine-woolled sheep from the ravages of the scab, which prevailed in the neighbourhood, and had already extended its pernicious influence to a flock of common sheep, belonging to M Flanders, of Espina. Another flock of the Merino's breed, belonging to the same gentleman, was submitted to vaccination, which produced its usual effect, and preserved the flock in the midst of the contagion. Forty of the sheep which had undergone the operation were placed among the infected flock, but they withstood the attacks of the disease, while not one of those which had not been vaccinated escaped.

---

*BILL*

### *BILL OF MORTALITY,*

### For Portsmouth, New Hampshire, for A. D. 1801.

### By LYMAN SPALDING, M.B. &c.

| COMPLAINT. | AGE. | Jan. | Feb. | Mar. | Apr. | May | June | July | Aug. | Sept | Oct. | Nov. | Dec. | Total. |
|---|---|---|---|---|---|---|---|---|---|---|---|---|---|---|
| Aphtha | 3 weeks | | | | | | | | 1 | | | | | 1 |
| Apoplexy | 39 years | | | | | | | | | | | | 1 | 1 |
| Atrophy | 3 weeks, 50, 3 years, 4 months, 55 years | 1 | | | | | 2 | | | 1 | | | 1 | 5 |
| Cancer | 65, 76 years | | | | | | | | 1 | | 1 | | | 2 |
| Cholera Infantum | 6 to 18 months | | | | | | | 1 | 1 | 2 | 3 | | | 7 |
| Consumption | 54, 50, 56, 75, 44, 35, 27, 83, 65, 69<br>56, 50, 60, 44, 32, 55, 26, 40, 48, 32 | 4 | 1 | | 1 | 3 | | 1 | 3 | 1 | 2 | 2 | 2 | 20 |
| Debauchery | 25, 29 years | | | | 1 | | | | 1 | | | | | 2 |
| Dropsy | 28, 41 years | | | | | | 1 | 1 | | | | | | 2 |
| Dropsy in the Brain | 12 months | | | 1 | | | | | | | | | | 1 |
| Epilepsy | 4 weeks, 10 years, 8 weeks | 1 | | 1 | | | | | 1 | | | | | 3 |
| Fever, Bilious | 26, 30, 45, 18, 28, 18, 14, 64, 8, 33 | | | 2 | | 1 | 2 | | | 2 | 1 | | 2 | 10 |
| Fever, Pulmonic | 12 days, 15, 21, 84 years | | 1 | | 1 | | | | | | 1 | | 1 | 4 |
| Hooping Cough | 3 months to 4 years | | | | | | | | | | 4 | 7 | | 11 |
| Illiac Passion | 95 years | | | | | | | | | | | 1 | | 1 |
| Mortification | 60 years | 1 | | | | | | | | | | | | 1 |
| Nephritis | 74, 66 years | | | | | | 1 | | | | 1 | | | 2 |
| Old Age | 82, 99, 75, 76, 80 | 2 | | | | | | 1 | | 1 | | 1 | | 5 |

| | | | | | | | | | | | | | | | |
|---|---|---|---|---|---|---|---|---|---|---|---|---|---|---|---|
| | Palsy | 54, 42, 68, 64, 77, 46, 64, 64, 43, 60, 19, 80 | | | 2 | 2 | | 3 | | | 2 | | | 3 | 12 |
| | Phrenitis | 31 years | | 1 | | | | | | | | | | | 1 |
| | Scrophula | 8 years | | | | 1 | | | | | | | | | 1 |
| | Still-born | | 1 | | | | | | | | | | | | 1 |
| *Casualties.* | Burnt | 71 years | | | | | | | | | | | | 1 | 1 |
| | Drowned | 60, 18, 45 years | | | | | | 1 | | | | | 1 | 1 | 3 |
| | Fall | 17 years | | | | | | | | | | 1 | | | 1 |
| | Frozen | 38 years | 1 | | | | | | | | | | | | 1 |
| | Paregoric | 6 months | | | | | | | | | | 1 | | | 1 |
| | | Total | 11 | 3 | 6 | 6 | 4 | 10 | 4 | 8 | 9 | 15 | 12 | 12 | 100 |

Portsmouth, situated 43 d. 5 m. north, 70 d. 41 m. west from London, contains about 5600 inhabitants. The town has been very healthful, not one in fifty-five having died. A bilious remitting fever prevailed the whole year, which in several instances, in September and October, manifested the malignant type. From June to October, the cholera infantum was prevalent. From September to the end of the year, the hooping cough was endemic, very few children escaped it. A fifth part have died of phthisis pulmonalis! "*Is there no balm in Gilead? Is there no physician there?*

M. DEYEUX

M. Deyeux has invented a new filter for purifying water. The substance through which the water passes is charcoal, in small pieces, but not reduced to powder. At the School of Medicine in Paris, he poured water taken from the kennel, and some in which putrid carcases had been immersed three weeks, upon his filtres, and in a few minutes it ran off in both cases perfectly clear, limpid, and without taste or smell.

---

Messrs. Harman and Dearn, of Rotherhithe, have invented an apparatus for filtering water, which will obviate the inconveniences of the filtering stone. The new apparatus consists of a stone ware vessel, perforated with holes upon which coarse gravel is laid, upon that a stratum of fine gravel, and lastly fine sand. Upon the top of the sand is laid a perforated and loaded board or plate of earthern ware, to prevent the sand from being disturbed when the water is poured in. The fineness and depth of the siliceous sand will regulate the perfection and expedition of the process, and the delicacy of the vessels and sand may be insured by changing the latter from time to time; for example, once in a fortnight or three weeks.

---

The following is a prize-question proposed at Paris:—"What are the characters that, in animal and vegetable matter, distinguish the active and passive substances in the operation of fermentations?"

---

Mr. Donovan, author of the Natural Histories of British Birds, Insects, &c. will shortly lay before the public the Natural History of British Shells, including coloured figures, arranged after the Linnean manner, with scientific and general Observations on each.

